# Evaluating the Rates of Pancreatitis and Pancreatic Cancer Among GLP‐1 Receptor Agonists: A Systematic Review and Meta‐Analysis of Randomised Controlled Trials

**DOI:** 10.1002/edm2.70113

**Published:** 2025-09-23

**Authors:** Jimmy Wen, Denise Nadora, Ethan Bernstein, Christiane How‐Volkman, Alina Truong, Bethany Joy, Megan Kou, Zohaer Muttalib, Arsh Alam, Eldo Frezza

**Affiliations:** ^1^ College of Medicine, California Northstate University Elk Grove California USA; ^2^ University of California Davis California USA

**Keywords:** GLP‐1 RA, pancreatic cancer, pancreatitis

## Abstract

**Aims:**

This meta‐analysis evaluates the rates of pancreatitis/pancreatic cancer among glucagon‐like peptide‐1 receptor agonists (GLP‐1 RAs) in randomised controlled trials (RCTs).

**Methods:**

Following the Preferred Reporting Items for Systematic Reviews and Meta‐Analyses (PRISMA), a systematic search was performed in PubMed, Embase, and Cochrane Library for GLP‐1 RA RCTs that evaluated pancreatitis/pancreatic cancer. A meta‐analysis was conducted to evaluate this risk; subgroup analysis was performed with and without background medications.

**Results:**

62 studies utilising dulaglutide, exenatide, liraglutide, semaglutide, beinaglutide, retatrutide, or tirzepatide, with 66,232 patients, mean age of 58.3 years (14.4 to 68), and mean follow‐up of 43.5 weeks (1 to 198) were included in this study. Meta‐analysis showed a significantly increased risk of pancreatitis (RR: 1.44, 95% CI 1.09–1.89, *p* = 0.009), but not when stratified by background medications (RR: 1.28, 95% CI 0.87–1.87) and without background medications (RR: 1.37, 95% CI 0.91–2.05). Pancreatic cancer and GLP‐1 RA use showed no significant association (RR: 1.30, 95% CI 0.86–1.97). However, a significant increase was found with background medications (RR: 1.85, 95% CI 1.05–3.26, *p* = 0.03), but not without (RR: 0.81, 95% CI 0.43–1.55).

**Conclusion:**

GLP‐1 RAs carry a slightly increased risk of pancreatitis, which is not significant when stratified by background medication use. Overall risk for pancreatic cancer was not observed, but a slight association was found when stratified with background medications. However, this difference is likely minimal, given the numerous studies excluded from the meta‐analysis where both treatment arms had zero events.

## Graphical abstract

The aim of this systematic review and meta‐analysis was to assess the risk of acute pancreatitis and pancreatic cancer among patients taking glucagon‐like peptide‐1 receptor agonists (GLP‐1 RAs). Meta‐analysis of 62 studies found a significantly increased risk of pancreatitis, but not when stratified with and without background medications.

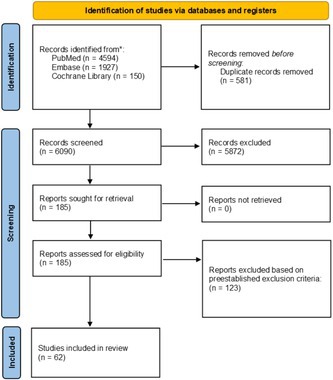

## 1 Introduction

The glucagon‐like peptide‐1 (GLP‐1) hormone is released in response to nutrient ingestion, enhancing insulin secretion, reducing glucagon release, and delaying gastric emptying [1]. Agonists of this hormone were identified as providing potential therapeutic benefits for patients with type 2 diabetes mellitus (T2DM), including improved glycaemic control and weight reduction [2]. In addition to glucose control, GLP‐1 receptor agonist (RA) therapy has evolved to address cardiovascular benefits, weight management, blood pressure, and cholesterol levels [3].

Despite these advantages, concerns were raised in preclinical models and case reports highlighting pancreatic changes and acute pancreatitis, respectively. Although the total number of cases was low, these initial findings led to the Food and Drug Administration (FDA) warnings in 2007 on the potential increased risk of pancreatitis [4, 5]. GLP‐1 RAs, such as exenatide, have been shown to induce pancreatic duct gland expansion and exacerbate chronic pancreatitis, potentially accelerating the formation of dysplastic lesions, including pancreatic intraepithelial neoplasia (PanIN), by increasing ductal cell proliferation and promoting acinar‐to‐ductal metaplasia [5, 6].

Findings of pancreatitis in these trials have been inconsistent, and subsequent studies have disputed the link between GLP‐1 RAs and pancreatitis. Clinical trials have monitored serum amylase and lipase, common markers of pancreatic health, and utilised independent adjudication committees to diagnose instances of pancreatitis to address these concerns [7]. This study aims to evaluate available clinical trials of different GLP‐1 RAs to identify the rates of pancreatitis/pancreatic cancer associated with their use. We hypothesise that GLP‐1 RAs will not significantly increase the risk of pancreatitis/pancreatic cancer.

## 2 Methods

### 2.1 Search Strategy

On May 6, 2024, a systematic search was performed following the guidelines from the Preferred Reporting Items for Systematic Reviews and Meta‐Analyses (PRISMA) framework. The following search query was utilised in PubMed, Embase, and Cochrane Library: ((pancrea*) OR (pancreatitis)) AND (((((((((glucagon‐like peptide‐1) OR (glp‐1)) OR (lixisenatide)) OR (liraglutide)) OR (semaglutide)) OR (tirzepatide)) OR (albiglutide)) OR (exenatide)) OR (dulaglutide)).

### 2.2 Article Selection Process

The Patient, Intervention, Comparison, Outcome, Time (PICOT) methodology was utilised to guide article selection. The patient population and intervention encompassed patients of any age taking a GLP‐1 RA. As all studies were randomised controlled trials (RCTs), comparisons with active comparators or placebo were included. The main outcomes assessed were rates of acute pancreatitis and pancreatic cancer among different agents within the GLP‐1 RA class. Studies with a minimum follow‐up of 1 week were included. The following inclusion criteria were utilised: (1) RCTs with patients taking GLP‐1 RAs; (2) active GLP‐1 RAs or discontinued GLP‐1 RAs (e.g., albiglutide) that were compared against another active GLP‐1 RA; (3) reported on adverse events (AEs) with a focus on pancreatitis or pancreatic cancer. Although we excluded studies that compared albiglutide or lixisenatide against placebo, we included studies that compared those agonists against other GLP‐1 RAs. These head‐to‐head trials were retained to provide comparative safety data for the active GLP‐1 RAs that are still in use, which remain clinically relevant for clinicians and patients. Exclusion criteria consisted of studies that did not meet the three inclusion criteria, discontinued GLP‐1 RAs (e.g., albiglutide) when compared against placebo, or if they were study designs such as non‐RCTs, case reports, review articles, expert opinions, abstracts, commentaries, animal studies, and studies not in English. This systematic review protocol is registered with PROSPERO as CRD42024550993.

Two independent authors participated in the title/abstract and full‐text screening of each article to determine study eligibility for inclusion. If there was a discrepancy between authors, the lead author (J.W.) reviewed the article for final study eligibility. Upon completion of full‐text screening, a rigorous reference search was performed for the included studies to assess for further studies to add to this review.

### 2.3 Study Quality and Risk of Bias

The risk of bias (RoB) and study quality was assessed utilising the Cochrane RoB tool, given that all included studies were RCTs. Two independent authors assessed the risk of bias and methodological quality, and any disagreements were resolved via rigorous re‐review until consensus was achieved. The Cochrane tool contains seven domains: sequence generation, allocation concealment, blinding of participants and personnel, blinding of outcomes, incomplete outcome data, selective outcome reporting, and other sources of bias. Each domain is evaluated as high, low, or unclear RoB. Two independent authors analysed each article, and any discrepancies were resolved via rigorous re‐review or by consulting a third reviewer until agreement was reached.

### 2.4 Data Extraction and Analysis

The study variables extracted and analysed included title, author, study year, number of patients, GLP‐1 RA agent and dosages, mean age, mean follow‐up time, pancreatitis/pancreatic cancer rates, related lab values (amylase/lipase), and those taking background anti‐diabetic drugs. Notably, the diagnostic criteria were not consistently reported across the studies and were reported as investigator or an independent committee–determined AE. The data was compiled using Google Sheets (Google Drive; Google, Mountain View, CA). The mean, median, percentages, ranges, and standard deviations were reported if available and applicable.

Meta‐analysis was conducted using the fixed effects model to evaluate the risk ratio (RR) of developing acute pancreatitis or pancreatic cancer. Chronic pancreatitis was not specified for pooling, and pancreatic cancer was an exploratory outcome, given that trial durations were not expected to be long enough to assess the risks for these two conditions. Sensitivity analyses were conducted with RCTs that had at least 24 weeks of follow‐up to test for differences in pancreatitis and pancreatic cancer findings. Heterogeneity was calculated with the *I*‐squared statistic, with *p*‐values less than 0.05 considered statistically significant. Forest plots were generated using Cochrane's Review Manager application (RevMan, version 5.4; The Cochrane Collaboration, London, UK). Studies with double‐zero events (treatment arms that had zero events) were included in the forest plot for transparency.

## 3 Results

**FIGURE 1:**
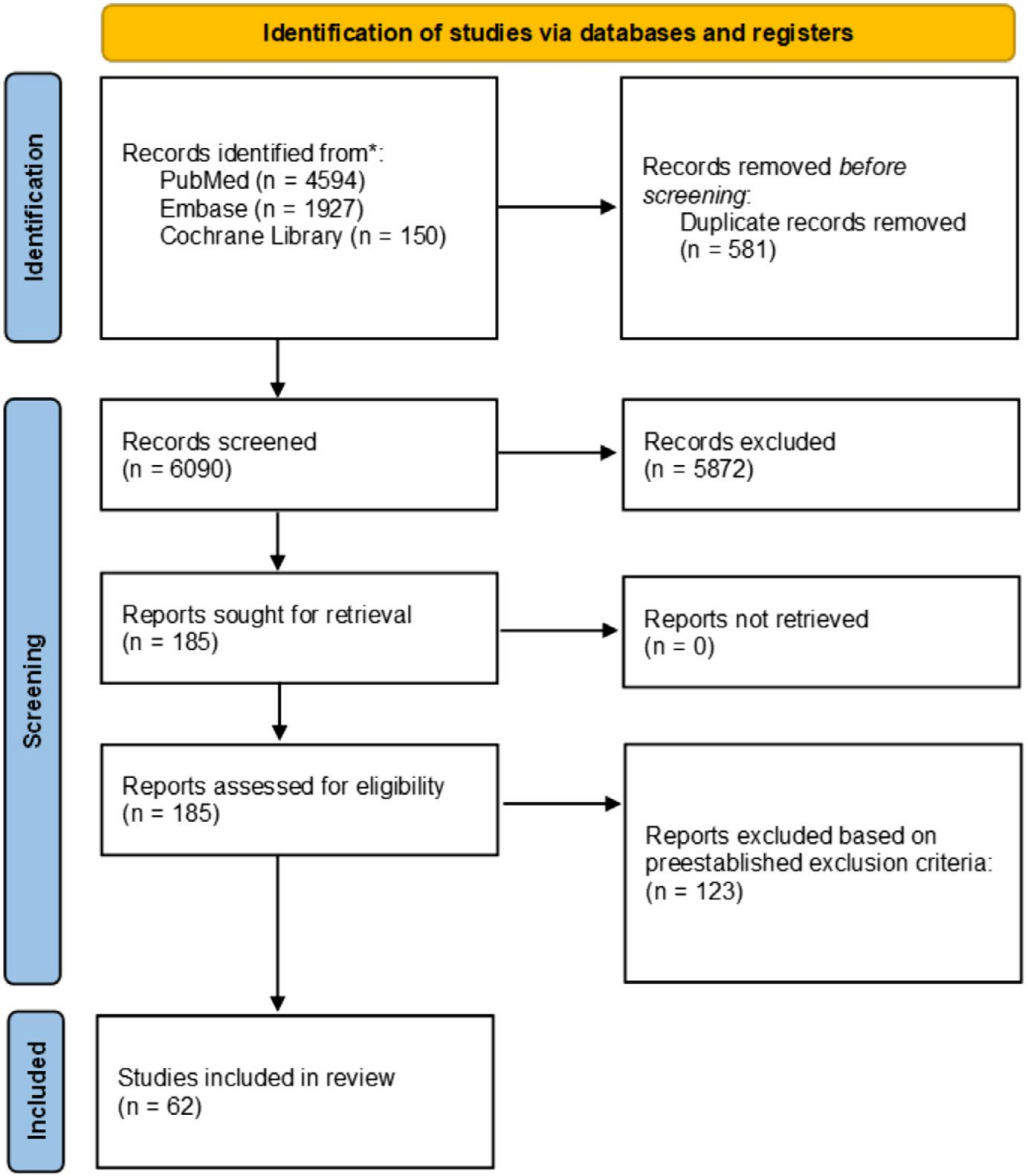
PRISMA Diagram of the included studies.

The initial search yielded 6671 articles from PubMed, Embase, and Cochrane Library. The articles were screened for duplicates and subsequently underwent title/abstract screening and full‐text review, which yielded 62 studies to be included in this systematic review. The screening process is further detailed in Figure 1.

### 3.1 Demographic Data

Across the 62 studies, there were a total of 66,232 patients (34,941 male/32,291 female) with a mean age of 58.3 years (14.4 to 68.0), mean duration of diabetes of 9.5 years (1.5 to 21.6), mean body mass index (BMI) of 32.6 kg/m^2^ (24.4 to 46.2), and mean follow‐up of 43.5 weeks (1 week to 198 weeks). (Table 1).

**TABLE 1:** Patient demographics.

Author, publication year	Number of patients enrolled	Sex (male, female)	Mean age in years (SD)	Mean follow‐up (weeks)	Duration of diabetes in years (SD)	BMI kg/m^2^ (SD)
Ahmann, 2015	Total: 450 Liraglutide: 225 Placebo: 225	Liraglutide: 120.0, 105.0 Placebo: 136.0, 89.0	Liraglutide: 59.3 (9.2) Placebo: 57.5 (11.1)	1	Liraglutide: 12.1 ± 7.1 Placebo: 12.1 ± 6.8	Liraglutide: 32.3 ± 5.6 Placebo: 32.2 ± 5.7
Ahren, 2016	Total: 831 Liraglutide 1.8 mg: 205 Liraglutide 1.2 mg: 209 Liraglutide 0.6 mg: 211 Placebo: 206	Liraglutide 1.8 mg: 92.0, 113.0 Liraglutide 1.2 mg: 102.0, 107.0 Liraglutide 0.6 mg: 93.0, 118.0 Placebo: 95.0, 111.0	Liraglutide 1.8 mg: 43.2 Liraglutide 1.2 mg: 42.8 Liraglutide 0.6 mg: 43.9 Placebo: 42.7	26	Liraglutide 1.8 mg: 21.4 Liraglutide 1.2 mg: 21.1 Liraglutide 0.6 mg: 21.0 Placebo: 20.7	Liraglutide 1.8 mg: 28.9 Liraglutide 1.2 mg: 28.8 Liraglutide 0.6 mg: 28.9 Placebo: 28.9
Ahrén, 2017	Total: 1225 Semaglutide 0.5 mg: 409 Semaglutide 1.0 mg: 409 Sitagliptin 100 mg: 407	Semaglutide 0.5 mg: 207.0, 202.0 Semaglutide 1.0 mg: 205.0, 204.0 Sitagliptin 100 mg: 208, 199.0	Semaglutide 0.5 mg: 54.8 (10.2) Semaglutide 1.0 mg: 56.0 (9.4) Sitagliptin 100 mg: 54.6 (10.4)	56	Semaglutide 0.5 mg: 6.4 (4.7) Semaglutide 1.0 mg: 6.7 (5.6) Sitagliptin 100 mg: 6.6 (5.1)	Semaglutide 0.5 mg: 32.4 (6.2) Semaglutide 1.0 mg: 32.5 (6.6) Sitagliptin 100 mg: 32.5 (5.8)
Araki, 2015	Total: 361 Dulaglutide: 181 Glargine: 180	Dulaglutide: 125.0, 56.0 Glargine: 133.0, 47.0	Dulaglutide: 57.5 Glargine: 56.1	26	Dulaglutide: 8.9 (6.7) Glargine: 8.8 (6.1)	Dulaglutide: 26.1 (3.6) Glargine: 25.9 (3.9)
Armstrong, 2016	Total: 52 Metabolic Dysfunction‐Associated Steatotic Liver Disease (MASLD) patients Liraglutide: 26 Placebo: 26	Liraglutide: 18.0, 8.0 Placebo: 13.0, 13.0	Liraglutide: 50.0 (11.0) Placebo: 52.0 (12.0)	48	Not reported (NR)	NR
Azar, 2016	Total: 341 Liraglutide: 171 Sulfonylurea (SU): 170	Liraglutide: 85.0, 86.0 SU: 83.0, 87.0	Liraglutide: 54.9 SU: 54.0	33	Liraglutide: 8.0 (5.3) SU: 7.2 (4.4)	Liraglutide: 30.2 (5.4) SU: 31.4 (5.9)
Bailey, 2016	Total: 406 Liraglutide: 202 Sitagliptin: 204	Liraglutide: 117.0, 85.0 Sitagliptin: 124.0, 80.0	Liraglutide: 56.3 (10.6) Sitagliptin: 56.5 (9.7)	26	Liraglutide: 7.9 (5.7) Sitagliptin: 7.6 (6.2)	Liraglutide: 31.7 (6.0) Sitagliptin: 32.2 (6.2)
Buse, 2009^a^	Total: 389	Liraglutide to Liraglutide to Liraglutide: 114.0, 119.0 Exenatide to Liraglutide to Liraglutide: 127.0, 104.0	Liraglutide to Liraglutide to Liraglutide: 56.3 (9.8) Exenatide to Liraglutide to Liraglutide: 57.1 (10.8)	40	Liraglutide to Liraglutide to Liraglutide: 8.5 (6.2) Exenatide to Liraglutide to Liraglutide: 7.9 (5.9)	Liraglutide to Liraglutide to Liraglutide: 32.9 (5.5) Exenatide to Liraglutide to Liraglutide: 32.9 (5.7)
Capehorn, 2020	Total: 577 Semaglutide: 290 Liraglutide: 287	Semaglutide: 160.0, 130.0 Liraglutide: 167.0, 120.0	Semaglutide: 60.1 (10.5) Liraglutide: 58.9 (10.0)	30	Semaglutide: 9.6 (6.1) Liraglutide: 8.9 (5.7)	Semaglutide: 33.7 (6.6) Liraglutide: 33.7 (7.0)
Chen, 2024	Total: 420 Beinaglutide: 282 Placebo: 138	Beinaglutide: 137.0, 145.0 Placebo: 65.0, 73.0	Beinaglutide: 35.3 (9.1) Placebo: 36.9 (8.7)	16	NR	Beinaglutide: 31.7 (4.6) Placebo: 31.5 (4.7)
Davies, 2015	Total: 846 Liraglutide 3 mg: 423 Liraglutide 1.8 mg: 211 Placebo: 212	Liraglutide 3 mg: 220.0, 203.0 Liraglutide 1.8 mg: 108.0, 103.0 Placebo: 97.0, 115.0	Liraglutide 3 mg: 55.0 (10.8) Liraglutide 1.8 mg: 54.9 (10.7) Placebo: 54.7 (9.8)	56	Liraglutide 3 mg: 7.5 (5.7) Liraglutide 1.8 mg: 7.4 (5.2) Placebo: 6.7 (5.1)	Liraglutide 3 mg: 37.1 (6.5) Liraglutide 1.8 mg: 37.0 (6.9) Placebo: 37.4 (7.1)
Davies, 2016	Total: 279 Liraglutide 1.8 mg: 140 Placebo: 139	Liraglutide: 75.0, 65.0 Placebo: 65.0, 72.0	Liraglutide: 68.0 (8.3) Placebo: 68.0 (8.3)	26	Liraglutide: 15.9 (8.9) Placebo: 14.2 (7.5)	Liraglutide: 33.4 (5.4) Placebo: 34.5 (5.4)
Drucker, 2008	Total: 293 2.0 mg exenatide QW: 148 10 μg exenatide BID: 145	2.0 mg Exenatide QW: 82.0, 66.0 10 μg exenatide BID: 75.0, 72.0	2.0 mg Exenatide QW: 55.0 (10.0) 10 μg exenatide BID: 55.0 (10.0)	30	2.0 mg Exenatide QW: 7.0 (6.0) 10 μg exenatide BID: 6.0 (5.0)	2.0 mg Exenatide QW: 35.0 (5.0) 10 μg exenatide BID: 35.0 (5.0)
Dungan, 2014	Total: 599 Dulaglutide: 299 Liraglutide: 300	Dulaglutide: 138.0, 161.0 Liraglutide: 149.0, 151.0	Dulaglutide: 56.5 (9.3) Liraglutide: 56.8 (9.9)	26	Dulaglutide: 7.1 (5.4) Liraglutide: 7.3 (5.4)	Dulaglutide: 33.5 (5.1) Liraglutide: 33.6 (5.2)
Dungan, 2016	Total: 299 Dulaglutide: 239 Placebo: 60	Dulaglutide: 104.0, 135.0 Placebo: 28.0, 32.0	Dulaglutide: 57.7 (10.2) Placebo: 58.2 (7.4)	24	Dulaglutide: 7.8 (5.3) Placebo: 6.8 (3.8)	Dulaglutide: 30.9 (5.2) Placebo: 32.4 (5.9)
Gerstein, 2019	Total: 9901 Dulaglutide: 4949 Placebo: 4952	Dulaglutide: 2643.0, 2306.0 Placebo: 2669.0, 2283.0	Dulaglutide: 66.2 (6.5) Placebo: 66.2 (6.5)	5.4	Dulaglutide: 10.5 (7.3) Placebo: 10.6 (7.2)	Dulaglutide: 32.3 (5.7) Placebo: 32.3 (5.8)
Giorgino, 2015	Total: 807 Dulaglutide 1.5 mg: 273 Dulaglutide 0.75 mg: 272 Glargine: 262	Dulaglutide 1.5 mg: 144.0, 129.0 Dulaglutide 0.75 mg: 136.0, 136.0 Glargine: 134.0,128.0	Dulaglutide 1.5 mg: 56.0 Dulaglutide 0.75 mg: 57.0 Glargine mg: 57.0	78	Dulaglutide 1.5 mg: 9.0 (6.0) Dulaglutide 0.75 mg: 9.0 (6.0) Glargine mg: 9.0 (6.0)	Dulaglutide 1.5 mg: 31.0 (5.0) Dulaglutide 0.75 mg: 32.0 (5.0) Glargine mg: 32.0 (6.0)
Holman, 2017	Total: 14752 Exenatide: 7356 Placebo: 7396	Exenatide: 4562.0, 2794.0 Placebo: 4587.0, 2809.0	Exenatide: 57.1 Placebo: 57.1	167	Total: 12.0	Total: 31.8
Inagaki, 2016	Total: 361 Dulaglutide: 181 Dulaglutide + SU: 34 Dulaglutide + SU+ Biguanide (BG): 83 Dulaglutide + BG: 64 Insulin Glargine (IG): 180 IG + SU: 33 IG + SU+ BG: 91 IG + BG: 66	Dulaglutide: 125.0, 56.0 DG + SU: 21.0,13.0 DG + SU + BG: 61.0, 22.0 DG + BG: 43.0, 21.0 IG: 133.0, 47.0 IG + SU: 29.0, 4.0 IG + SU + BG: 56.0, 25.0 IG + BG: 48.0, 18.0	DG + SU: 60.7 DG + SU + BG: 57 DG + BG: 56.5 IG + SU: 57.8 IG + SU + BG: 56.3 IG + BG: 55.1	26	DG + SU: 8.5 (6.3) DG + SU + BG: 10.8 (7.4) DG + BG: 6.7 (5.2) IG + SU: 8.7 (5.5) IG + SU + BG: 9.8 (6.0) IG + BG: 7.6 (6.2)	DG + SU: 24.9 (3.4) DG + SU + BG: 26.4 (3.5) DG + BG: 26.3 (3.6) IG + SU: 24.6 (3.1) IG + SU + BG: 25.8 (4.2) IG + BG: 26.5 (3.8)
Ji, 2013	Total: 678 Exenatide QW: 340 Exenatide BID: 338	Exenatide QW: 183.0, 157.0 Exenatide BID: 184.0, 154.0	55.5	26	Total: > 7	Exenatide QW: 26.4 Exenatide BID: 26.7
Kelly, 2020	Total: 251 Liraglutide: 125 Placebo: 126	Liraglutide: 71.0, 54.0 Placebo: 48.0, 78.0	Liraglutide: 14.6 Placebo: 14.5	56	NR	Liraglutide: 35.3 (5.1) Placebo: 35.8 (5.7)
Klausen, 2023	Total 127 Exenatide: 62 Placebo: 65	Exenatide: 3.0, 25.0 Placebo: 39.0, 26.0	Exenatide: 52.1 (10.8) Placebo: 52.5 (10.0)	26	NR	Exenatide: 26.7 (5.2) Placebo: 26.7 (4.6)
Klein, 2014	Total: 21 Liraglutide: 14	Liraglutide: 5.0, 9.0 Placebo: 2.0, 5.0	Liraglutide: 14.4 Placebo: 15.6	5	Liraglutide: 1.7 Placebo: 1.7	Liraglutide: 40.0 Placebo: 39.9
Kuchay, 2020	Total: 64 MASLD patients Dulaglutide: 32 Placebo: 32	Dulaglutide: 23.0, 9.0 Control: 22.0, 10.0	Dulaglutide: 46.6 Control: 48.1	24	Dulaglutide: 4.9 (3.1) Control: 5.7 (4.3)	Dulaglutide: 29.6 (3.6) Control: 29.9 (3.9)
Lau, 2021	Total: 706 Cagrilinitide 0.3 mg: 101 Cagrilinitide 0.6 mg: 100 Cagrilinitide 1.2 mg: 102 Cagrilinitide 2.4 mg: 102 Cagrilinitide 4.5 mg: 101 Liraglutide 3.0 mg: 99 Placebo: 101	Cagrilinitide 0.3 mg: 45.0, 56.0 Cagrilinitide 0.6 mg: 38.0, 62.0 Cagrilinitide 1.2 mg: 39.0, 63.0 Cagrilinitide 2.4 mg: 27.0, 75.0 Cagrilinitide 4.5 mg: 45.0, 56.0 Liraglutide 3.0 mg: 34.0, 65.0 Placebo: 42.0, 59.0	Cagrilinitide 0.3 mg: 53.5 Cagrilinitide 0.6 mg: 53.2 Cagrilinitide 1.2 mg: 52.1 Cagrilinitide 2.4 mg: 52.7 Cagrilinitide 4.5 mg: 51.5 Liraglutide 3.0 mg: 51.5 Placebo: 51.4	26	NR	Cagrilinitide 0.3 mg: 38.4 (7.5) Cagrilinitide 0.6 mg: 37.2 (6.9) Cagrilinitide 1.2 mg: 37.1 (6.2) Cagrilinitide 2.4 mg: 37.9 (7.6) Cagrilinitide 4.5 mg: 38.4 (7.7) Liraglutide 3.0 mg: 38.4 (7.4) Placebo: 37.4 (5.7)
LeRoux, 2017	Total 2254 Liraglutide: 1505 Placebo: 749	Liraglutide: 364.0, 1141.0 Placebo: 176.0, 573.0	Liraglutide: 47.5 Placebo: 47.3	160	NR	Liraglutide: 38.8 (6.4) Placebo: 39.0 (6.3)
Marso, 2016	Total: 3297 Total Semaglutide: 1648 Semaglutide 0.5 mg: 826 Semaglutide 1.0 mg: 822 Total Placebo: 1649 Placebo 0.5 mg: 824 Placebo 1.0 mg: 825	Semaglutide 0.5 mg: 495.0, 331.0 Semaglutide 1.0 mg: 518.0, 304.0 Placebo 0.5 mg: 482.0, 342.0 Placebo 1.0 mg: 507.0, 318.0	Semaglutide 0.5 mg: 64.6 Semaglutide 1.0 mg: 64.7 Placebo 0.5 mg: 64.8 Placebo 1.0 mg: 64.4	104	Semaglutide 0.5 mg: 14.3 (8.2) Semaglutide 1.0 mg: 14.1 (8.2) Placebo 0.5 mg: 14.0 (8.5) Placebo 1.0 mg: 13.2 (7.4)	Semaglutide 0.5 mg: 32.7 (6.3) Semaglutide 1.0 mg: 32.9 (6.2) Placebo 0.5 mg: 32.9 (6.4) Placebo 1.0 mg: 32.7 (6.0)
Marso, 2016	Total: 9340 Liraglutide 1.8 mg: 4668 Placebo: 4672	Liraglutide: 425.0, 183.0 Placebo: 485.0, 209.0	Liraglutide: 64.3 Placebo: 64.4	198	Liraglutide: 12.8 (8.0) Placebo: 12.9 (8.1)	Liraglutide: 32.5 (6.3) Placebo: 32.5 (6.3)
Mathieu 2016	Total: 1389 Liraglutide 1.8 mg: 346 Liraglutide 1.2 mg: 346 Liraglutide 0.6 mg: 350 Placebo: 347	Liraglutide 1.8 mg: 165.0, 181.0 Liraglutide 1.2 mg: 167.0, 179.0 Liraglutide 0.6 mg: 164.0, 186.0 Placebo: 169.0, 180.0	Liraglutide 1.8 mg: 43.7 (13.3) Liraglutide 1.2 mg: 43.9 (13.1) Liraglutide 0.6 mg: 43.6 (12.8) Placebo: 43.4 (12.6)	52	Liraglutide 1.8 mg: 21.5 (12.6) Liraglutide 1.2 mg: 21.6 (12.2) Liraglutide 0.6 mg: 20.9 (12.2) Placebo: 21.6 (11.8)	Liraglutide 1.8 mg: 29.5 (5.2) Liraglutide 1.2 mg: 29.3 (5.1) Liraglutide 0.6 mg: 29.5 (5.3) Placebo: 29.8 (5.6)
Meier, 2015	Total: 142 Lixisenatide 20 μg: 48 Liraglutide 1.2 mg: 47 Liraglutide 1.8 mg: 47	Lixisenatide 20 μg: 33.0,15.0 Liraglutide 1.2 mg: 39.0, 8.0 Liraglutide 1.8 mg: 33.0, 14.0	Lixisenatide 20 μg: 61.6 Liraglutide 1.2 mg: 61.4 Liraglutide 1.8 mg: 62.6	8	Lixisenatide 20 μg: 11.4 Liraglutide 1.2 mg: 10.5 Liraglutide 1.8 mg: 12.5	Lixisenatide 20 μg: 30.7 (4.3) Liraglutide 1.2 mg: 30.5 (4.0) Liraglutide 1.8 mg: 31.2 (4.3)
Miras, 2019	Total: 80 Liraglutide: 53 Placebo: 27	Liraglutide 20.0, 33.0 Placebo: 13.0, 14.0	Liraglutide: 55.0 Placebo: 57.0	26	Liraglutide 16.4 (7.0) Placebo: 19.6 (8.0)	Liraglutide 36.1 (7.8) Placebo: 37.0 (7.7)
Miyagawa, 2015	Total: 492 Dulaglutide 0.75 mg: 281 Liraglutide 0.9 mg: 141 Placebo: 70	Dulaglutide: 228.0, 52.0 Liraglutide: 113.0, 24.0 Placebo: 55.0, 15.0	Dulaglutide: 57.2 (9.6) Liraglutide: 57.9 (10.4) Placebo: 57.7 (8.3)	26	Dulaglutide: 6.8 (5.6) Liraglutide: 6.3 (6.0) Placebo: 6.3 (5.1)	Dulaglutide: 25.6 (3.6) Liraglutide: 25.5 (3.5) Placebo: 25.2 (3.2)
Myat, 2021	Total: 22 Saline then Liraglutide: 12 Liraglutide then saline: 10	Saline to liraglutide: 11.0, 1.0 Liraglutide tosaline: 9.0,1.0	Saline then liraglutide: 65.3 Liraglutide then saline: 59.2	6	NR	Saline then liraglutide: 30.1 (3.4) Liraglutide then saline: 29.6 (4.8)
Nauck, 2014	Total: 1038 Dulaglutide 1.5 mg: 304 Dulaglutide 0.75 mg: 302 Sitagliptin: 315 Placebo: 177	Dulaglutide 1.5 mg: 146.0, 158.0 Dulaglutide 0.75 mg: 134.0, 168.0 Sitagliptin: 151.0, 164.0 Placebo: 90.0, 87.0	Dulaglutide 1.5 mg: 54.0 Dulaglutide 0.75 mg: 54.0 Sitagliptin: 54.0 Placebo: 55.0	52	Dulaglutide 1.5 mg: 7.0 (6.0) Dulaglutide 0.75 mg: 7.0 (5.0) Sitagliptin: 7.0 (5.0) Placebo: 7.0 (5.0)	Dulaglutide 1.5 mg: 31.0 (5.0) Dulaglutide 0.75 mg: 31.0 (4.0) Sitagliptin: 31.0 (4.0) Placebo: 31.0 (4.0)
Nauck, 2012	Total: 411 Semaglutide 0.1 mg: 47 Semaglutide 0.2 mg: 43 Semaglutide 0.4 mg: 48 Semaglutide 0.8 mg: 42 Semaglutide 0.8 mg dose escalation (E): 43 Semaglutide 1.6 E: 47 Liraglutide 1.2 mg: 45 Liraglutide 1.8 mg: 50 Placebo: 46	Semaglutide 0.1 mg: 31.0,16.0 Semaglutide 0.2 mg: 30.0, 13.0 Semaglutide 0.4 mg: 37.0, 11.0 Semaglutide 0.8 mg: 22.0, 20.0 Semaglutide 0.8 mg E: 27.0, 16.0 Semaglutide 1.6 E: 26.0, 21.0 Liraglutide 1.2 mg: 31.0, 14.0 Liraglutide 1.8 mg: 35.0, 15.0 Placebo: 28.0, 18.0	Semaglutide 0.1 mg: 55.2 (10.1) Semaglutide 0.2 mg: 54.7 (10.0) Semaglutide 0.4 mg: 53.8 (10.2) Semaglutide 0.8 mg: 55.0 (9.7) Semaglutide 0.8 mg E: 55.9 (7.9) Semaglutide 1.6 mg E: 56.4 (10.5) Liraglutide 1.2 mg: 54.8 (9.2) Liraglutide 1.8 mg: 54.3 (10.1) Placebo: 55.3 (10.6)	12	Semaglutide 0.1 mg: 3.6 (5.0) Semaglutide 0.2 mg: 2.3 (2.7) Semaglutide 0.4 mg: 2.0 (2.3) Semaglutide 0.8 mg: 3.0 (3.0) Semaglutide 0.8 mg E: 2.6 (2.1) Semaglutide 1.6 mg E: 1.8 (2.0) Liraglutide 1.2 mg: 3.3 (3.4) Liraglutide 1.8 mg: 2.5 (2.6) Placebo: 2.4 (3.3)	Semaglutide 0.1 mg: 31.5 (4.6) Semaglutide 0.2 mg: 30.4 (3.9) Semaglutide 0.4 mg: 29.7 (4.5) Semaglutide 0.8 mg: 30.7 (4.5) Semaglutide 0.8 mg E: 31.2 (4.2) Semaglutide 1.6 mg E: 30.9 (4.7) Liraglutide 1.2 mg: 31.0 (4.6) Liraglutide 1.8 mg: 30.9 (4.6) Placebo: 31.7 (3.8)
Papamargaritis, 2024	Total: 392 Control: 132 Liraglutide 3 mg: 260	Control: 53.0, 79.0 Liraglutide: 87.0, 173.0	Control: 51.8 Liraglutide: 51.1	104	NR	Control: 45.5 (7.3) Liraglutide: 46.2 (7.8)
Pi‐Sunyer, 2015	Total: 3731 Liraglutide 3.0 mg: 2487 Placebo: 1244	Liraglutide: 530.0, 1957.0 Placebo: 273.0, 971.0	Liraglutide: 45.2 (12.1) Placebo: 45.0 (12.0)	56	NR	Liraglutide: 38.3 (6.4) Placebo: 38.3 (6.3)
Pozzilli, 2017	Total: 300 Dulaglutide 1.5 mg: 150 Placebo: 150	Dulaglutide 1.5 mg: 85.0, 65.0 Placebo: 88.0, 62.0	Dulaglutide 1.5 mg: 60.2 (9.5) Placebo: 60.6 (10.1)	28	Dulaglutide 1.5 mg: 13.0 (7.5) Placebo: 13.3 (7.7)	Dulaglutide 1.5 mg: 32.8 (4.9) Placebo: 32.6 (4.9)
Pratley, 2012	Total: 436 Sitagliptin to liraglutide: 135 Liraglutide only: 284	Sitagliptin to liraglutide: 67.0, 68.0 Liraglutide only: 144.0, 140.0	Sitagliptin to liraglutide: 54.2 Liraglutide only: 55.1	78	NR	NR
Pratley, 2010	Total: 665 Liraglutide 1.2 mg: 225 Liraglutide 1.8 mg: 221 Sitagliptin: 219	Liraglutide 1.2 mg: 116.0, 109.0 Liraglutide 1.8 mg: 116.0, 105.0 Sitagliptin: 120.0, 99.0	Liraglutide 1.2 mg: 55.9 (9.6) Liraglutide 1.8 mg: 55 (9.1) Sitagliptin: 55.0 (9.0)	26	Liraglutide 1.2 mg: 6.0 (4.5) Liraglutide 1.8 mg: 6.4 (5.4) Sitagliptin: 6.3 (5.4)	Liraglutide 1.2 mg: 32.6 (5.2) Liraglutide 1.8 mg: 33.1 (5.1) Sitagliptin: 32.6 (5.4)
Retnakaran, 2014	Total: 51 Liraglutide: 25 Placebo: 26	Liraglutide: 16.0, 10.0 Placebo: 16.0, 9.0	Liraglutide: 58.9 (8.7) Placebo: 57.4 (7.4)	48	Liraglutide: 3.0 Placebo: 1.5	Liraglutide: 30.0 (4.3) Placebo: 30.4 (5.8)
Rosenstock, 2023	Total: 281 Placebo: 45 Retatrutide 0.5 mg: 47 Retatrutide 4 mg escalation: 23 Retatrutide 4 mg: 24 Retatrutide 8 mg slow escalation: 26 Retatrutide 8 mg fast escalation: 46 Retatrutide 12 mg escalation: 24 Dulaglutide 1.5 mg: 46	Placebo: 22.0, 23.0 Retatrutide 0.5 mg: 24.0, 23.0 Retatrutide 4 mg escalation group: 15.0, 8.0 Retatrutide 4 mg group: 12.0, 12.0 Retatrutide 8 mg slow escalation group: 10.0, 16.0 Retatrutide 8 mg fast escalation group: 9.0, 15.0 Retatrutide 12 mg escalation group: 20.0, 26.0 Dulaglutide 1.5 mg: 13.0, 33.0	Placebo: 57.6 (10.8) Retatrutide 0.5 mg: 57.2 (9.7) Retatrutide 4 mg escalation group: 57.7 (8.1) Retatrutide 4 mg group: 57.6 (10.0) Retatrutide 8 mg slow escalation group: 57.0 (7.4) Retatrutide 8 mg fast escalation group: 53.8 (9.0) Retatrutide 12 mg escalation group: 54.4 (9.7) Dulaglutide 1.5 mg: 54.9 (10.4)	36	Placebo: 8.7 (8.3) Retatrutide 0.5 mg: 8.8 (6.7) Retatrutide 4 mg escalation group: 8.1 (6.6) Retatrutide 4 mg group: 10.5 (7.6) Retatrutide 8 mg slow escalation group: 7.2 (6.4) Retatrutide 8 mg fast escalation group: 6.0 (5.8) Retatrutide 12 mg escalation group: 7.9 (6.9) Dulaglutide 1.5 mg: 7.2 (6.5)	Placebo: 33.8 (4.9) Retatrutide 0.5 mg: 34.7 (5.6) Retatrutide 4 mg escalation group: 36.3 (7.4) Retatrutide 4 mg group: 34.0 (6.5) Retatrutide 8 mg slow escalation group: 35.0 (6.4) Retatrutide 8 mg fast escalation group: 34.1 (5.9) Retatrutide 12 mg escalation group: 35.5 (6.9) Dulaglutide 1.5 mg: 36.3 (6.8)
Rosenstock, 2017	Total: 460 ITCA 650 40 ug/day: 153 ITCA 650 60 ug/day: 153 Placebo: 154	ITCA 650 40 ug/day: 89.0, 64.0 ITCA 650 60 ug/day: 91.0, 62.0 Placebo: 92.0, 62.0	ITCA 650 40 ug/day: 55.5 (10.3) ITCA 650 60 ug/day: 54.7 (9.6) Placebo: 54.7 (9.1)	26	ITCA 650 40 ug/day: 9.1 (6.2) ITCA 650 60 ug/day: 8.9 (6.9) Placebo: 8.6 (6.0)	ITCA 650 40 ug/day: 33.1 (5.1) ITCA 650 60 ug/day: 33.8 (5.2) Placebo: 33.7 (5.5)
Rosenstock, 2009	Total: 84 Exenatide BID 5–10 μg: 34 Placebo: 50	NR	54.0	16	Total: 4.9	NR
Rubino, 2022	Total: 338 Semaglutide: 126 Liraglutide: 127 Placebo: 86	Semaglutide 2.4 mg: 24.0, 102.0 Liraglutide 3.0 mg: 30.0, 97.0 Placebo: 19.0, 66.0	Semaglutide: 48.0 Liraglutide: 49.0 Placebo: 51.0	68	NR	Semaglutide: 37.0 (7.4) Liraglutide: 37.2 (6.4) Placebo: 38.8 (6.5)
Santilli, 2017	Total: 40 Liraglutide: 20 Lifestyle changes: 20	Liraglutide: 11.0, 9.0 Lifestyle changes: 10.0, 10.0	Liraglutide: 55.5 Lifestyle changes: 52.2	17.4	NR	Liraglutide: 36.7 Lifestyle changes: 35.0
Seino, 2010	Liraglutide: 268 Glibenclamide: 132	Liraglutide: 182.0, 86.0 Glibenclamide: 89.0, 43.0	Liraglutide: 58.2 (10.4) Glibenclamide: 58.5 (10.4)	12	Liraglutide: 8.1 (6.7) Glibenclamide: 8.5 (6.8)	Liraglutide: 24.5 (3.7) Glibenclamide: 24.4 (3.8)
Siskind, 2018	Total: 28 Exenatide: 14 Placebo: 14	Exenatide: 11.0, 3.0 Placebo: 7.0, 7.0	NR	25	NR	Exenatide: 35.6 (2.4) Placebo: 35.8 (3.8)
Smits, 2017	Total: 55 Liraglutide: 19 Sitagliptin: 19 Placebo: 17	Liraglutide: 14.0, 5.0 Sitagliptin: 16.0, 3.0 Placebo: 13.0, 4.0	Liraglutide: 60.5 (7.2) Sitagliptin: 61.7 (6.8) Placebo: 65.8 (5.8)	12	Liraglutide: 7.7 (4.5) Sitagliptin: 8.1 (5.8) Placebo: 8.2 (4.8)	Liraglutide: 33.3 (4.5) Sitagliptin: 31.5 (4.3) Placebo: 30.6 (2.9)
Sorli, 2017	Total: 387 Semaglutide 0.5 mg: 128 Semaglutide 1.0 mg: 130 Placebo: 129	Semaglutide 0.5 mg: 60.0, 68.0 Semaglutide 1.0 mg: 80.0, 50.0 Placebo: 70.0, 59.0	Semaglutide 0.5 mg: 54.6 (11.1) Semaglutide 1.0 mg: 52.7 (11.9) Placebo: 53.9 (11.0)	30	Semaglutide 0.5 mg: 4.8 (6.1) Semaglutide 1.0 mg: 3.6 (4.9) Placebo: 4.1 (5.5)	Semaglutide 0.5 mg: 32.5 (7.6) Semaglutide 1.0 mg: 33.9 (8.4) Placebo: 32.4 (6.9)
Tanaka, 2015	Total: 47 Metformin: 24 Liraglutide: 22	Metformin: 16.0, 8.0 Liraglutide: 13.0, 9.0	Metformin: 51.0 (11.0) Liraglutide: 55.0 (11.0)	24	Metformin: 4.7 (3.9) Liraglutide: 5.6 (4.2)	Metformin: 28.7 (3.7) Liraglutide: 28.6 (4.2)
Terauchi, 2014	Total: 145 Dulaglutide 0.25 mg: 36 Dulaglutide 0.5 mg: 37 Dulaglutide 0.75 mg: 35 Placebo: 37	Dulaglutide 0.25 mg: 27.0, 9.0 Dulaglutide 0.5 mg: 23.0, 14.0 Dulaglutide 0.75 mg: 28.0, 7.0 Placebo: 29.0, 8.0	Dulaglutide 0.25 mg: 52.3 (8.8) Dulaglutide 0.5 mg: 52.5 (9.2) Dulaglutide 0.75 mg: 52.2 (7.8) Placebo: 51.7 (9.7)	12	Dulaglutide 0.25 mg: 4.3 (3.5) Dulaglutide 0.5 mg: 4.9 (4.0) Dulaglutide 0.75 mg: 4.6 (4.5) Placebo: 4.7 (4.5)	Dulaglutide 0.25 mg: 26.8 (4.5) Dulaglutide 0.5 mg: 26.7 (3.8) Dulaglutide 0.75 mg: 27.1 (3.7) Placebo: 27.4 (4.5)
Tuttle, 2018	Total: 577 Dulaglutide 1.5 mg: 193 Dulaglutide 0.75 mg: 190 Glargine: 194	Dulaglutide: 1.5 mg: 104.0, 88.0 Dulaglutide: 0.75 mg: 104.0, 86.0 Glargine: 93.0, 101.0	Dulaglutide 1.5 mg: 64.7 (8.8) Dulaglutide 0.75 mg: 64.7 (8.6) Glargine: 64.3 (8.4)	52	Dulaglutide 1.5 mg: 17.6 (8.7) Dulaglutide 0.75 mg: 18.0 (8.8) Glargine: 18.7 (8.7)	Dulaglutide 1.5 mg: 32.1 (4.8) Dulaglutide 0.75 mg: 33.0 (5.5) Glargine: 32.4 (5.3)
Umpierrez, 2011	Total: 262.0 Dulaglutide (0.5/1.0): 66.0 Dulaglutide (1.0/1.0): 65.0 Dulaglutide (1.0/2.0): 65.0 Placebo: 66.0	Dulaglutide (0.5/1.0): 35.0, 31.0 Dulaglutide (1.0/1.0): 35.0, 30.0 Dulaglutide (1.0/2.0): 34.0, 31.0 Placebo: 29.0, 37.0	Dulaglutide 0.5/1.0 mg: 59.0 (12.0) Dulaglutide 1.0/1.0 mg: 57.0 (12.0) Dulaglutide 1.0/2.0 mg: 54.0 (11.0) Placebo: 56.0 (12.0)	16	Dulaglutide LY 0.5/1.0 mg: 9.0 (7.6) Dulaglutide LY 1.0/1.0 mg: 8.1 (5.4) Dulaglutide LY 1.0/2.0 mg: 8.6 (6.9) Placebo: 7.5 (5.4)	Dulaglutide LY 0.5/1.0 mg: 33.7 (4.1) Dulaglutide LY 1.0/1.0 mg: 33.9 (4.0) Dulaglutide LY 1.0/2.0 mg: 34.2 (4.1) Placebo: 33.9 (4.3)
vanRaalte, 2016	Total: 36 Exenatide: 16 Glargine: 20	Exenatide: 11.0, 5.0 Glargine: 14.0, 6.0	Exenatide: 58.0 (1.0) Glargine: 58.0 (1.0)	156	Exenatide: 5.7 (0.8) Glargine: 4.0 (0.6)	Exenatide: 30.9 (0.7) Glargine: 30.1 (0.6)
Wadden, 2013	Total: 422 Liraglutide: 212 Placebo: 210	Liraglutide: 34.0, 178.0 Placebo: 44.0, 166.0	Liraglutide: 45.9 (11.9) Placebo: 46.5 (11)	56	NR	Liraglutide: 38.2 (6.2) Placebo: 37.5 (6.2)
Wadden, 2023	Total: 579 Tirzepatide: 287 Placebo: 292	Tirzepatide: 106.0, 181.0 Placebo: 109.0, 183.0	Tirzepatide: 45.4 (12.6) Placebo 45.7 (11.8)	72	NR	Tirzepatide: 38.7 (6.6) Placebo 38.4 (6.8)
Wägner, 2019	Total: 24 Liraglutide: 12 Placebo: 12	Liraglutide: 5.0, 7.0 Placebo: 4.0, 8.0	Liraglutide 53.2 (9.7) Placebo: 52.6 (13.8)	26	Liraglutide 10.0 (7.2) Placebo: 7.4 (4.1)	Liraglutide 34.1 (6.6) Placebo: 35.9 (6.0)
Wilding, 2021	Total: 1961 Semaglutide: 1306 Placebo: 655	Semaglutide: 351.0, 955.0 Placebo: 157.0, 498.0	Semaglutide: 46.0 (13.0) Placebo: 47.0 (12.0)	68	NR	Semaglutide: 37.8 (6.7) Placebo: 38.0 (6.5)
Xu, 2015	Total: 416 Exenatide: 110 Insulin: 114 Pioglitazone: 118	Exenatide: 74.0, 36.0 Insulin: 70.0, 44.0 Pioglitazone: 65.0, 53.0	NR	48	NR	Exenatide: 25.9 (0.3) Insulin: 25.4 (0.3) Pioglitazone: 25.9 (0.3)
Zinman, 2009	Total: 533 Liraglutide 1.2 mg: 178 Liraglutide 1.8 mg: 178 Placebo: 177	Liraglutide 1.2: 101.0, 77.0 Liraglutide 1.8: 91.0, 87.0 Placebo: 110.0, 67.0	Liraglutide 1.2 mg: 55.0 (10.0) Liraglutide 1.8 mg: 55.0 (11.0) Placebo: 55.0 (10.0)	26	Liraglutide 1.2 mg: 9.0 (6.0) Liraglutide 1.8 mg: 9.0 (6.0) Placebo: 9.0 (6.0)	Liraglutide 1.2 mg: 33.2 (5.4) Liraglutide 1.8 mg: 33.5 (5.1) Placebo: 33.9 (5.2)
Zinman, 2007	Total: 233 Exenatide: 121 Placebo: 112	Exenatide: 65.0, 56.0 Placebo: 64.0, 48.0	Exenatide: 55.6 (10.8) Placebo: 56.6 (10.2)	16	Exenatide: 7.3 (4.9) Placebo: 8.2 (5.8)	Exenatide: 34.0 (5.1) Placebo: 34.0 (5.0)

^a^ Study by Buse 2009 is an extension study; demographic information is from the initial study.

The systematic review includes 62 RCTs comparing various GLP‐1 RAs, including various dosages of dulaglutide, exenatide, liraglutide, semaglutide, beinaglutide, retatrutide, and tirzepatide. The review also divides studies between those that included patients who continued background medications to maintain glycaemic control and those that did not use background medications. These medications included any combination of metformin, sulfonylureas, insulin, dipeptidyl peptidase 4 (DPP‐4) inhibitors, thiazolidinediones, Sodium‐Glucose Cotransporter 2 inhibitor (SGLT‐2i), and other background anti‐diabetic medication except GLP‐1 RAs.

### 3.2 Study Characteristics

Across the seven domains, most studies were low risk of bias for sequence generation (50/62), incomplete outcome data (54/62), selective outcome reporting (60/62), and other sources of bias (33/62). However, allocation concealment had a substantial amount of unclear ranking (29/62), and high risks of bias were found for blinding of participants/personnel (20/62) as well as blinding of outcomes (23/62). These results are depicted in Tables S1 and S2.

### 3.3 Pancreatitis and Pancreatic Cancer

Across all studies that included patients who continued background medications, the greatest number of pancreatitis and pancreatic cancer occurred in patients who received dulaglutide [28 (0.35%) and 19 (0.24%), respectively]. Across the studies that included patients who did not receive background medications, the greatest number of pancreatitis occurred in patients who received exenatide [42 (0.56%)] and the greatest number of pancreatic cancer occurred in patients who received dulaglutide [1 (0.93%)]. These results are illustrated in Table 2.

**TABLE 2:** Number of pancreatic events in patients without background medications.

GLP‐1 RA	Author, year	Pancreatic AE (*n*)^a^
Beinaglutide	Chen 2024	0
Dulaglutide 0.25 mg, 0.5 mg, 0.75 mg	Terauchi 2014	Pancreatic cancer: 1
Exenatide	Holman 2017	41
Exenatide	Klausen 2023	0
Exenatide	Siskind 2018	0
Exenatide	vanRaalte 2016	0
Exenatide	Xu 2015	1
Liraglutide	Kelly 2020	1
Liraglutide	Lau 2021	1
Liraglutide	LeRoux 2017	10
Liraglutide	Myat 2021	0
Liraglutide	Pi‐Sunyer 2015	10
Liraglutide	Retnakaran 2014	0
Liraglutide	Tanaka 2015	0
Liraglutide	Wadden 2013	0
Liraglutide Semaglutide	Rubino 2022	Liraglutide: 1 Semaglutide: 0
Semaglutide 0.5 mg, 1.0 mg	Marso 2016	Semaglutide 0.5 mg: 9 Semaglutide 1.0 mg: 4
Semaglutide 0.5 mg, 1.0 mg	Sorli 2017	0
Semaglutide	Wilding 2021	3
Tirzepatide 10 mg, 15 mg	Wadden 2023	Tirzepatide: 1 Placebo: 1

^a^ AE refers to the total number of pancreatitis events unless otherwise specified.

**FIGURE 2:**
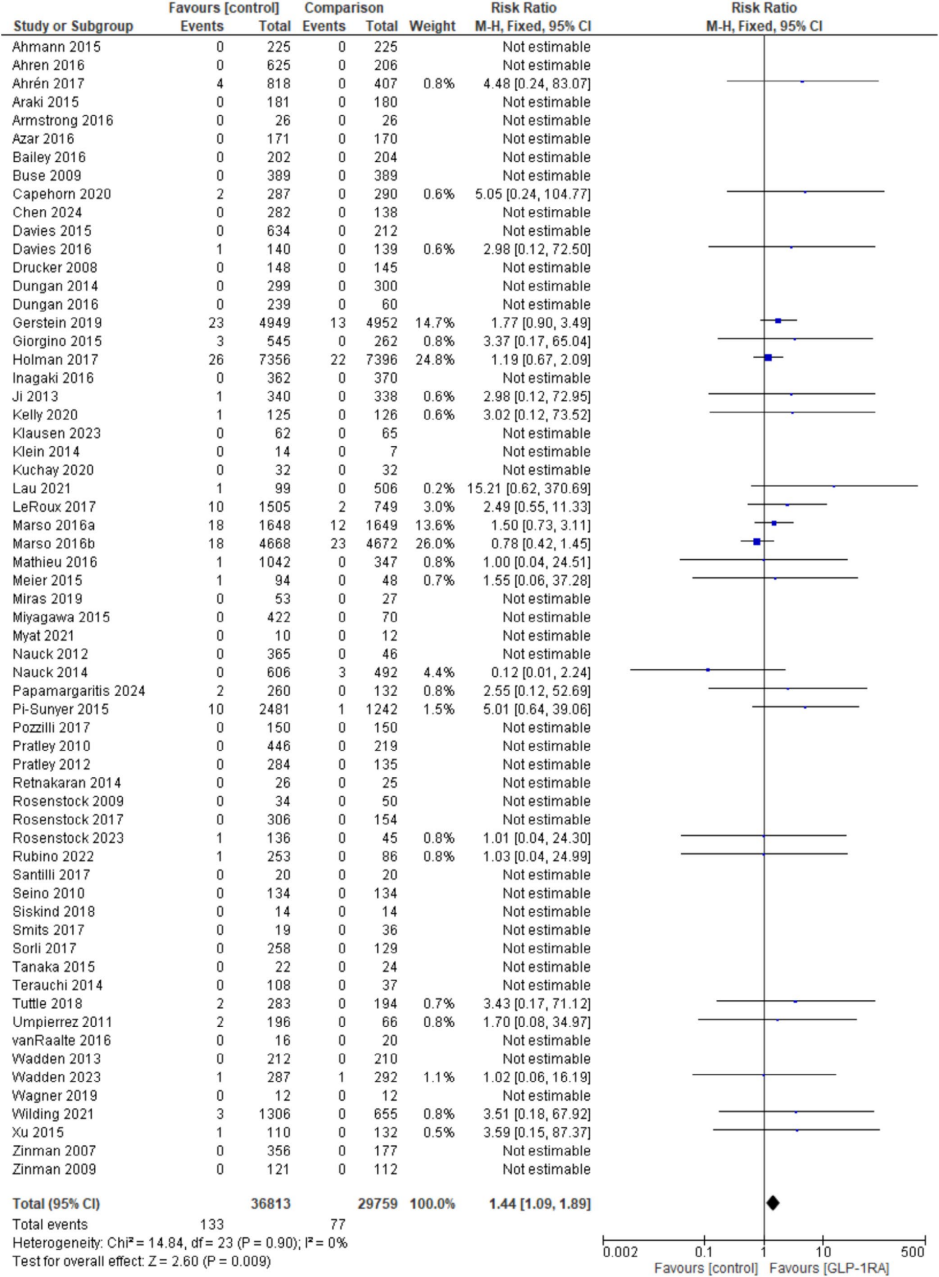
Meta‐analysis of GLP‐1 RA versus control for comparison of pancreatitis incidence.

A comprehensive meta‐analysis was conducted to assess the risk of pancreatitis and pancreatic cancer associated with GLP‐1 RAs compared to controls. In the pooled analysis of all studies evaluating pancreatitis incidence (Figure 2), GLP‐1 RA use was associated with a statistically significant increase in risk (RR = 1.44; 95% CI: 1.09–1.89; *p* = 0.009; *I* ^2^ = 0%). When stratified by background medication use, studies that included background therapies (Figure S1) and those that did not (Figure S2) both showed nonsignificant associations (RR = 1.28, 95% CI: 0.87–1.87, *p* = 0.22, *I* ^2^ = 0% and RR = 1.37, 95% CI: 0.91–2.05, *p* = 0.13, *I* ^2^ = 0%, respectively).

For pancreatic cancer, the overall pooled analysis (Figure S3) demonstrated no significant association with GLP‐1 RA use (RR = 1.30, 95% CI: 0.86–1.97, *p* = 0.22, *I* ^2^ = 0%). There was also no significant association found in the subgroup analysis without background medications (Figure S4; RR = 0.81, 95% CI: 0.43–1.55, *p* = 0.53, *I* ^2^ = 0%); however, the results were significant with background medications (Figure S5; RR = 1.85; 95% CI: 1.05–3.26, *p* = 0.03, *I* ^2^ = 0%).

When subgrouped by studies that had a minimum 24‐week follow‐up, pancreatitis risk (RR = 1.43, 95% CI: 1.09–1.89, *p* = 0.01, *I* ^2^ = 0%) and pancreatic cancer (RR = 1.28, 95% CI: 0.84–1.96, *p* = 0.25, *I* ^2^ = 25%) remained statistically significant and non‐significant, respectively (Figures S6 and S7).

## 4 Discussion

This systematic review and meta‐analysis analysed 62 studies, with a total of 66,232 patients at a mean follow‐up of 43.5 weeks, to evaluate the rates of pancreatitis and pancreatic cancer among patients taking GLP‐1 RAs. The main findings of this study were that GLP‐1 RAs had a small but significant association with pancreatitis, but no significance with pancreatic cancer. However, subgroup analysis with and without background medications showed no association with pancreatitis. Notably, the inclusion of tirzepatide and retatrutide, which are dual GLP‐1/glucose‐dependent insulinotropic polypeptide (GIP) and triple GLP‐1/GIP/glucagon agonists, respectively, may influence the safety profile due to the additional receptor activation. However, the inclusion of these agents did not substantially alter the rates of the pooled effect estimate, but the results should be interpreted with this in mind.

Our study builds on previous analyses by incorporating the largest RCT dataset to date, which evaluated acute pancreatitis and pancreatic cancer. We also conducted subgroup analyses that stratified by background medications and study duration, allowing more focused risk evaluation of GLP‐1 RAs. Additionally, controlled trial data provide greater context for pharmacovigilance and post‐marketing safety data. All patients included in this analysis had long‐standing T2DM and were on stable anti‐hyperglycaemic medication before starting the screening process and study period. However, 45 of the 66 studies included in this study had patients who continued their medications throughout the study period, in addition to the GLP‐1 RA intervention (Table 3). Subgroup analyses showed no significant risk of pancreatitis among patients who had concomitant background medications and patients who did not, which indicates that background medications did not confound the relationship between GLP‐1 RA usage and pancreatitis. Although there was a significant risk found for pancreatic cancer among patients who had concomitant background medications, given the very small number of studies in the analyses, this finding must be interpreted with extreme caution. Furthermore, it is important to note that RCTs generally exclude patients who have a history of pancreatitis or risk factors. For example, only two of the included studies had patients with Metabolic Dysfunction‐Associated Steatotic Liver Disease (MASLD), which is noteworthy given the possible association in modulating pancreatic disease risk [1]. This limits the generalisability of these findings, as higher‐risk patients that would be seen in practice would be underrepresented in these study populations.

**TABLE 3:** Number of pancreatic events in patients with background medications.

GLP‐1 RA	Background medication	Author	Pancreatic AE^a^
Albiglutide Liraglutide	Metformin alone or metformin and sulfonylureas	Pratley 2014	Albiglutide: 1 Liraglutide: 2
Albiglutide Weekly 4 mg, Albiglutide Weekly 15 mg, Albiglutide Weekly 30 mg, Albiglutide Biweekly 15 mg, Albiglutide Biweekly 30 mg, Albiglutide Biweekly 50 mg, Albiglutide Monthly 50 mg, Albiglutide Monthly 100 mg, Exenatide twice daily 5–10 μg	Metformin	Rosenstock 2009	0
Dulaglutide	Sulfonylureas, biguanides, or both	Araki 2015	0
Dulaglutide 1.5 OW LIraglutide uptitrated from 0.6 mg/day to 1.2 mg/day to 1.8 mg/day	Metformin	Dungan 2014	0
Dulaglutide	Sulfonylurea	Dungan 2016	0
Dulaglutide	Metformin, sulfonylurea, insulin, DPP‐4 inhibitor, Thiazolidinedione or other glucose‐lowering drugs (not GLP1 receptor agonist or pramlintide)	Gerstein 2019	Pancreatitis: Dulaglutide: 23 Placebo: 13 Pancreatic cancer: Dulaglutide: 19 Placebo: 12
Dulaglutide 1.5 mg Dulaglutide 0.75 mg	Insulin glargine and/or sulfonylurea	Giorgino 2015	Dulaglutide 1.5 mg: 2 Dulaglutide 0.75 mg: 1
Dulaglutide	Sulfonylurea or biguanides or both	Inagaki 2016	0
Dulaglutide	Metformin, DPP‐4 inhibitors, sulfonylurea, and or insulin	Kuchay 2020	0
Dulaglutide 1.5 mg Dulaglutide 0.75 mg	Metformin and other oral anti‐diabetic medication (OAM)	Nauck 2014	Dulaglutide: 0 Sitagliptin group: 2 Placebo: 1
Dulaglutide	Glargine	Pozzilli 2017	0
Dulaglutide 1.5 mg Dulaglutide 0.75 mg	Insulin lispro	Tuttle 2018	Dulaglutide 1.5 mg: 2
Dulaglutide Liraglutide	Patients were allowed to initiate rescue therapy for severe, persistent hyperglycemia according to predefined thresholds on fasting blood glucose for at least 2 weeks with no readily identifiable cause.	Miyagawa 2015	Pancreatic cancer: Liraglutide: 1
Dulaglutide 1.5 mg Retatrutide 0.5 mg Retatrutide 4 mg escalation group Retatrutide 4 mg group, Retatrutide 8 mg slow escalation group Retatrutide 8 mg fast escalation group Retatrutide 12 mg escalation group	Metformin	Rosenstock 2023	Retatrutide 0.5 mg group: 1 Retatrutide 8 mg slow escalation group: 1
Exenatide 2.0 mg OW; 5 μg twice a day for the first 28 days, then 10 μg twice a day for the remainder of the 30‐week	Metformin only, sulfonylurea only, thiazolidinedione only, Metformin and sulfonylurea, metformin and thiazolidinedione, Metformin^b^, sulfonylurea^b^, thiazolidinedione^b^ – Sulfonylurea^b^: 54 (37%) – Thiazolidinedione^b^: 25 (17%)	Drucker 2008	0
Exenatide extended release wmg Once weekly Exenatide BID 10 μg	Metformin and/or sulfonylurea	Ji 2013	Extended release: 1
Exenatide	Metformin only, SU only, Metformin and SU, Metformin and SU and TZD	Rosenstock 2017	0
Exenatide	TZD and Metformin, TZD only	Zinman 2007	0
Liraglutide	Basal insulin analogue and/or metformin	Ahmann 2015	0
Liraglutide 0.6, 1.2, or 1.8 mg	Insulin	Ahren 2016	0
Liraglutide	Metformin, sulfonylurea	Armstrong 2016	0
LIraglutide	Metformin	Azar 2016	0
Liraglutide	Metformin	Bailey 2016	0
Liraglutide	Background oral antidiabetic drugs remained unchanged, although sulfonylurea doses could be decreased by 50% if unacceptable hypoglycemia occurred	Buse 2009	0
Liraglutide 1.8 mg or 3.0 mg	Metformin only, Metformin and glitazone, Metformin and sulfonylurea, Metformin and sulfonylurea and glitazone, Sulfonylurea only, or Sulfonylurea and glitazone	Davies 2015	0
Liraglutide	Insulin, sulfonylurea	Davies 2016	1
Liraglutide	Metformin (if previously treated with it)	Klein 2014	0
Liraglutide	Metformin, sulfonylureas, insulin (allowed but not for GLP‐1 agonists, DPP‐4 inhibitors, pramlintide)	Marso 2016	Pancreatitis: Liraglutide: 18 Placebo: 23 Pancreatic cancer: Liraglutide: 13 Placebo: 5
Liraglutide 0.6 mg, 1.2 mg, or 1.8 mg	Insulin	Mathieu 2016	Liraglutide 0.6 mg: 1
Liraglutide	Metformin or long‐acting insulin	Miras 2019	0
Liraglutide	Any combination of lifestyle, metformin, sulfonylureas, thiazolidinediones or sodium glucose co‐transporters‐2	Papamargaritis 2024	Liraglutide: 1
Liraglutide	Metformin	Pratley 2010	0
Liraglutide	Metformin	Pratley 2012	0
Liraglutide	Metformin	Santilli 2017	0
Liraglutide	Metformin with or without sulfonylurea	Smits 2017	0
Liraglutide	Oral agents (metformin, sulfonylurea) and/or intermediate or long‐acting insulin	Wägner 2019	0
Liraglutide	TZD+ metformin	Zinman 2009	0
Liraglutide 0.6 mg daily for 1 week then 1.2 mg once daily until end of trial. Liraglutide 1.8 mg Lixisenatide 10 μg once daily for 2 weeks followed by Lixisenatide 20 μg once daily for the remainder of the trial.	Metformin, insulin glargine	Meier 2015	Liraglutide: 1
Liraglutide 0.6 mg Semaglutide 0.25 mg	Biguanides, Sulfonylurea, SGLT‐2i, DPP‐4ib, Other blood glucose‐lowering drugs, excluding insulin	Capehorn 2019	Liraglutide: 2
Liraglutide 1.2 mg, or 1.8 mg Semaglutide 0.1 mg, 0.2 mg, 0.4 mg, 0.8 mg, 0.8 mg E, or 1.6 E	Metformin or on semaglutide	Nauck 2012	0
Lixisenatide	Insulin glargine and metformin	Riddle 2013	Placebo: 1
Lixisenatide Liraglutide	Metformin, glimepiride, intensified insulin therapy, basal insulin therapy	Quast 2021	0
LY 0.5 mg for 4 weeks then 1.0 mg for 12 weeks (LY 0.5/1.0); LY 1.0 mg for 16 weeks (LY 1.0/1.0); LY 1.0 mg for 4 weeks then 2.0 mg for 12 weeks (LY 1.0/2.0)	OAM from each of the two different classes (sulfonylurea, biguanide, thiazolidinedione or DPP‐IV inhibitors)	Umpierrez 2011	Dulaglutide: 2
Semaglutide 0.5 mg, 1.0 mg	Metformin, thiazolidinediones, or both	Ahrén 2017	Semaglutide 0.5 mg acute pancreatitis: 3 Semaglutide 1.0 mg chronic pancreatitis: 1

^a^ AE refers to the total number of pancreatitis events unless otherwise specified.

^b^ Specified agent alone or in combination.

The studies reviewed in this systematic analysis focused on patients classified as overweight or obese, with inclusion criteria encompassing individuals with a BMI ranging from 25 to 45. Amylase and lipase levels were also measured in some of the studies, with many patients showing elevated levels of these enzymes at the latest follow‐up. However, in some cases, despite the elevated levels, the increases were insufficient to meet the researchers' diagnostic criteria for pancreatitis.

This variation within the included studies limits the generalizability and validity of the pooled estimates. The low I^2^ heterogeneity is also likely due to the fixed‐effects model and the sparse number of events, rather than true homogeneity across the studies. Concomitant background medication usage may influence the observed risk of acute pancreatitis or pancreatic cancer, either through confounding or diluting the risk attributable to GLP‐1 RAs [8, 9, 10, 11]. For pancreatic cancer, subgroup analysis with background medications showed an association with pancreatic cancer. However, it is important to note that only studies with events were included in the pooled risk ratio estimate, as the other RCTs had zero events in both arms. This may overestimate the risk as the double‐zero studies would likely have diluted the RR, indicating that the true risk is lower than expected. Nevertheless, these zero‐event studies highlight the overall rare incidence of pancreatitis and pancreatic cancer in both arms. All studies were retained in the forest plots for transparency but were not included in the meta‐analysis.

### 4.1 Potential Mechanisms and Reported Rates in the Literature

Incretins have been suspected to relate to pancreatitis, especially as T2DM is associated with the development of acute and chronic pancreatitis [12]. Although the mechanism of action is not yet elucidated, warnings and precautions of pancreatitis are included in the prescribing information for GLP‐1 RAs. Preliminary studies have found that GLP‐1 elicits a beta‐cell preserving effect, and these sustained proliferative actions may cause unintentional exocrine and endocrine effects (e.g., pancreatitis, pancreatic cancer) [13]. Acute pancreatitis, specifically fatal haemorrhagic and necrotising types, has been noted, but the causal relationship between GLP‐1 RAs and pancreatitis/pancreatic carcinoma is not yet confirmed [14]. GLP‐1 RAs, thus, are not recommended to be prescribed for patients with a history of pancreatitis and should be discontinued if pancreatitis symptoms develop. However, recent literature has suggested that GLP‐1 RAs can be used in patients with a history of acute pancreatitis, albeit on an individualised basis where the glycaemic, cardiovascular, and weight loss benefits outweigh the risks [15]. A retrospective review of 161 patients with a history of acute pancreatitis at a mean follow‐up of 28.2 months found a total of 16 (9.9%) secondary episodes, of which 6 (37.5%) were attributed to the GLP‐1 RA [16]. The 9.9% rate is lower than a pooled recurrent pancreatitis rate by Li et al. of 21% (95% CI: 18%–24%) and a pooled incidence rate calculated by Gagyi et al. of 5.26 per 100 person‐years (CI: 3.99–6.94) [17, 18].

Previous studies utilised postmarketing reports and observational studies, which carry inherent limitations that decrease the strengths of those studies. Notably, among the database studies, it was found that there was remarkable notoriety bias for the AEs reported [19]. It is important to note that obesity increases the rates of pancreatitis and pancreatic cancer risk factors (e.g., gallstones, hypertriglyceridemia, biliary disease). Furthermore, since T2DM is a risk factor for pancreatic disease, it can be difficult to differentiate whether pancreatitis or pancreatic cancer is attributable to the underlying condition or the GLP‐1 RA. Thus, patient and treatment selection provide another source of imbalance and confounding across these studies. Other systematic reviews and meta‐analyses found no significant findings for pancreatitis or pancreatic cancer. Cao et al. collected data from seven cardiovascular outcome trials with 56,004 patients with T2DM and a median follow‐up ranging from 1.3 to 5.4 years, with no significant difference compared to placebo for acute pancreatitis (OR: 1.05, 0.78–1.40, *p* = 0.76) or pancreatic cancer (OR: 1.12, 0.77–1.63, *p* = 0.56) [19]. Similarly, a 2023 meta‐analysis with 43 trials found that pancreatitis (OR: 1.24, 0.94–1.64, *p* = 0.13) and pancreatic cancer (OR: 1.28, 0.87–1.89, *p* = 0.20) displayed no clear association for GLP‐1 RA usage [20]. Most recently, a 2024 population‐based cohort study following 543,595 patients at over 7 years of follow‐up supported no increased risk for pancreatic cancer (OR: 0.50, 0.15–1.71) [21]. However, an evaluation of the FDA Adverse Event Reporting System (FAERS) database from 2004 to 2020 found a significantly increased (proportional reporting ratio: 9.86) pancreatic cancer cases associated with GLP‐1 RAs compared to the other glucose‐lowering agents analysed in the study [22]. Notably, the authors stated that the combination of GLP‐1 RAs with DPP‐4 inhibitors may have led to an increased reporting of several tumours, including pancreatic cancer [22]. Interestingly, a 2024 study presented at the Endocrine Society's annual meeting evaluating 638,501 patients with a history of acute pancreatitis suggested that GLP‐1 RAs reduce the risk of recurrent acute pancreatitis [23]. However, these findings have not been published, and further research is required to detail these preliminary results. Regardless, this possibility will offer more options for patients that will help improve patient outcomes and quality of life.

### 4.2 Limitations

However, these findings must be understood within the context of their limitations. First, as mentioned earlier, there was heterogeneity in the included studies in terms of GLP‐1 RA dosage/type, study design, age (range of 14.4 to 68 years), follow‐up time (range of 1 to 198 weeks), and background medication usage. Most importantly, this heterogeneity was seen in the definition of pancreatitis, which was not consistently reported and was determined through investigator or independent committees. Second, we included only RCTs, which provide high‐quality evidence but generally have a shorter duration, which may not be long enough to fully capture the risk of pancreatic cancer. In contrast, databases and pharmacovigilance, such as FAERS, suggest increased pancreatic cancer risk, but these data are subject to reporting bias, preventing the determination of causality. Thus, the discrepancy between FAERS and the findings of this study highlights the need for long‐term observational studies to further clarify this risk. Third, a key limitation of the meta‐analysis is the exclusion of studies with zero events in both arms from the pooled risk ratio calculations. This was done due to the risk ratio becoming undefined if no events occur in either arm; these studies would not provide data to the pooled estimate and were excluded. However, this limits the comprehensiveness of the pooled values and can overestimate the overall relative risk as the excluded trials likely could dilute the observed risk ratio. RevMan software prevented smaller decimal precision of very small effect sizes, as we originally intended to use risk difference calculation, which could better capture the low incidence across all included studies. Fourth, despite the high quality of RCTs, patients typically enrolled in RCTs are highly selected and may not be representative of the general T2DM or obese population, possibly limiting the external validation of these findings. Fifth, although we included trials with at least a 1‐week follow‐up to maximise the capture of acute events of pancreatitis, this would limit the detection of longer‐term outcomes such as chronic pancreatitis and pancreatic cancer. Long‐term observational studies, real‐world evidence, or pharmacovigilance are needed to reliably test these outcomes. To test this, we conducted a sensitivity analysis with trials that had at least 12 weeks of follow‐up to assess for differences in pancreatitis. Sixth, patients with a history of pancreatitis or risk factors for pancreatitis are usually excluded during the screening phase. It is important to note that acute pancreatitis secondary to drugs is a diagnosis of exclusion. Thus, cases that are deemed idiopathic may very well be due to drug exposure as well. Additionally, a lack of classic symptoms or a lack of evolution of symptoms may lead to further underdiagnosis.

## 5 Conclusion

Overall analysis suggests a potential increased risk of pancreatitis with GLP‐1 RA use, which weakens when stratifying by background medication use. No conclusive evidence links GLP‐1 RA use to an increased risk of pancreatic cancer, but a slightly significant association was found when stratified with background medications. Clinicians should remain cautious in patients with a high baseline pancreatic risk, and GLP‐1 RA treatment should be discontinued if pancreatitis symptoms develop. Further research should address standardising GLP‐1 RA exposure, follow‐up protocols, patient characteristics, and background therapies to eliminate heterogeneity across trials.

## Author Contributions

The author takes full responsibility for this article.

## Conflicts of Interest

The authors declare no conflicts of interest.

## Supporting information


**Figure S1:** Meta‐analysis of GLP‐1 RA versus control for comparison of pancreatitis incidence (excluding studies w/o background medications).
**Figure S2:** Meta‐analysis of GLP‐1 RA versus control for comparison of pancreatitis incidence (ONLY studies w/o background medications).
**Figure S3:** Meta‐analysis of GLP‐1 RA versus control for comparison of pancreatic cancer incidence.
**Figure S4:** Meta‐analysis of GLP‐1 RA versus control for comparison of pancreatic cancer incidence (excluding studies w/o background medications).
**Figure S5:** Meta‐analysis of GLP‐1 RA versus control for comparison of pancreatic cancer incidence (ONLY studies w/o background medications).
**Figure S6:** Sensitivity Analysis Minimum 24‐Weeks of GLP‐1 RA versus control for comparison of pancreatitis incidence.
**Figure S7:** Sensitivity Analysis Minimum 24‐Weeks of GLP‐1 RA versus control for comparison of pancreatic cancer incidence.

**Table S1:** Cochrane Risk of Bias.
**Table S2:** Summary of Cochrane Risk of Bias.

## Data Availability

The datasets used and/or analysed in the current study are available upon reasonable request. Please contact J.W. to request data from the study.
