# Supplementary material for: Evaluating the Rates of Pancreatitis and Pancreatic Cancer Among GLP‐1 Receptor Agonists: A Systematic Review and Meta‐Analysis of Randomised Controlled Trials
Source: Endocrinol Diabetes Metab. 2025 Sep 23;8(5):e70113. doi: 10.1002/edm2.70113 (PMC12457091; doi:10.1002/edm2.70113)
Supplement: Supplementary file 1 — Figure S1: Meta‐analysis of GLP‐1 RA versus control for comparison of pancreatitis incidence (excluding studies w/o background medications). Figure S2: Meta‐analysis of GLP‐1 RA versus control for comparison of pancreatitis incidence (ONLY studies w/o background medications). Figure S3: Meta‐analysis of GLP‐1 RA versus control for comparison of pancreatic cancer incidence. Figure S4: Meta‐analysis of GLP‐1 RA versus control for comparison of pancreatic cancer incidence (excluding studies w/o background medications). Figure S5: Meta‐analysis of GLP‐1 RA versus control for comparison of pancreatic cancer incidence (ONLY studies w/o background medications). Figure S6: Sensitivity Analysis Minimum 24‐Weeks of GLP‐1 RA versus control for comparison of pancreatitis incidence. Figure S7: Sensitivity Analysis Minimum 24‐Weeks of GLP‐1 RA versus control for comparison of pancreatic cancer incidence. [file EDM2-8-e70113-s001.docx]

**
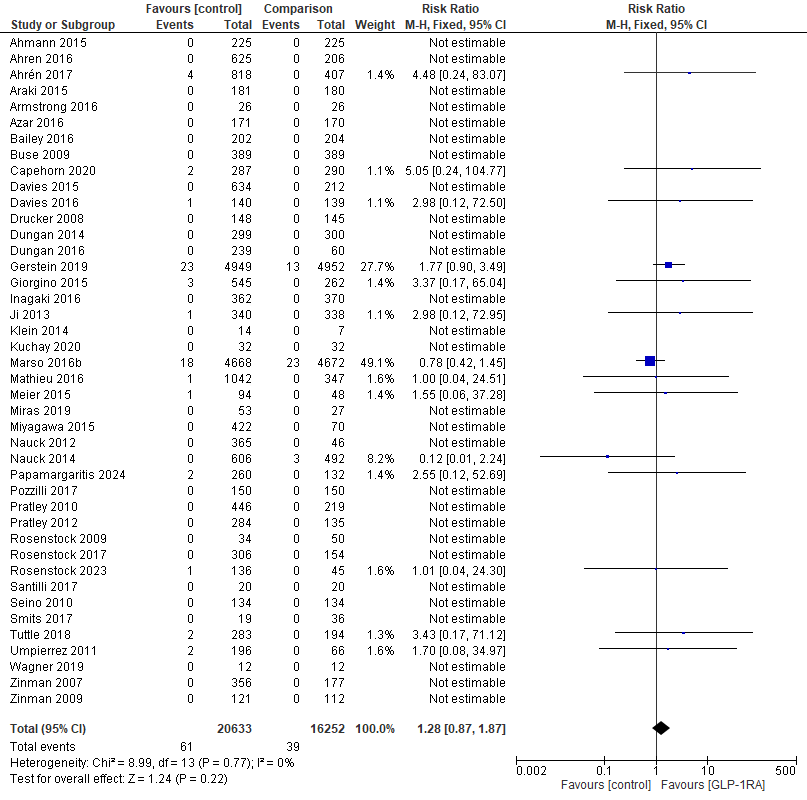
**

**Figure S1. Meta-analysis of GLP-1RA versus control for comparison of pancreatitis incidence (excluding studies w/o background medications)**

**
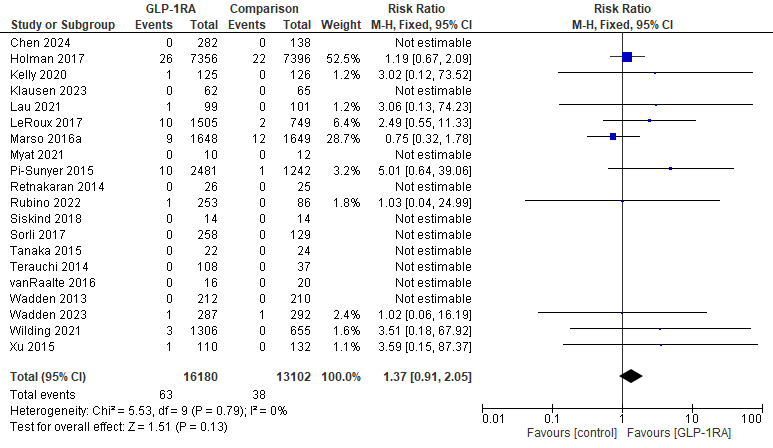
**

**Figure S2. Meta-analysis of GLP-1RA versus control for comparison of pancreatitis incidence (ONLY studies w/o background medications)**


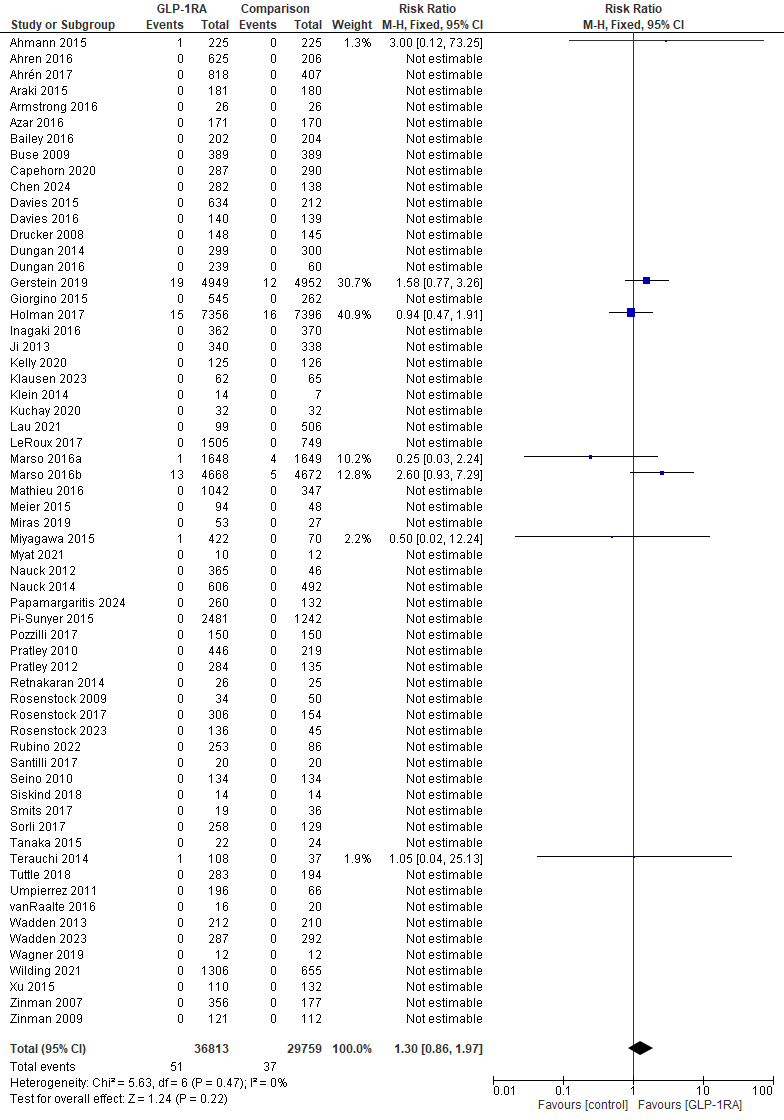


**Figure S3. Meta-analysis of GLP-1RA versus control for comparison of pancreatic cancer incidence (all)**


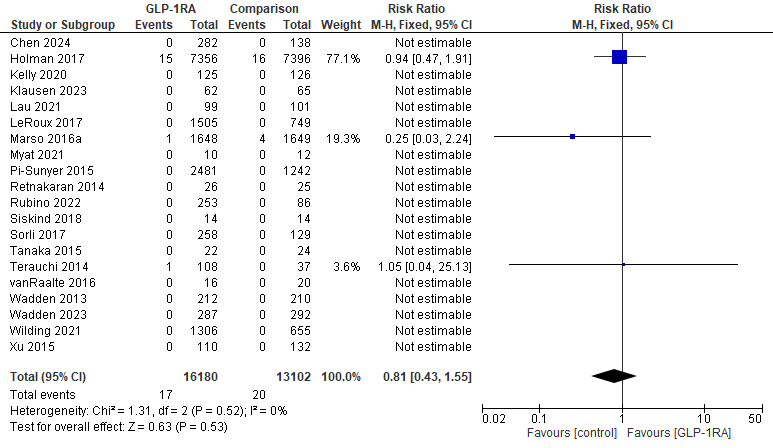


**Figure S4. Meta-analysis of GLP-1RA versus control for comparison of pancreatic cancer incidence (ONLY studies w/o background medications)**


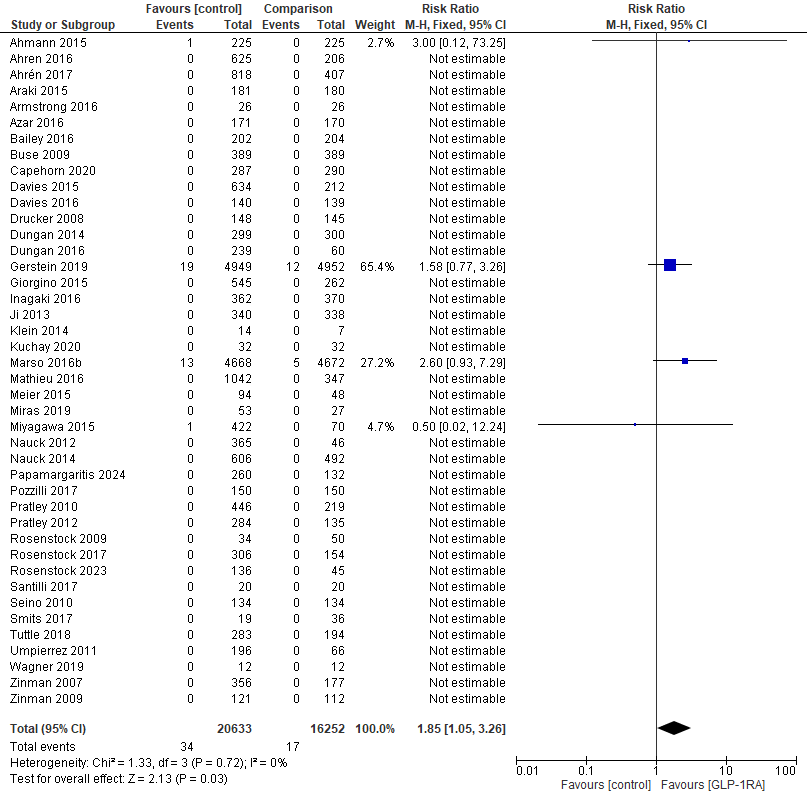


**Figure S5. Meta-analysis of GLP-1RA versus control for comparison of pancreatic cancer incidence (excluding studies w/o background medications)**

**
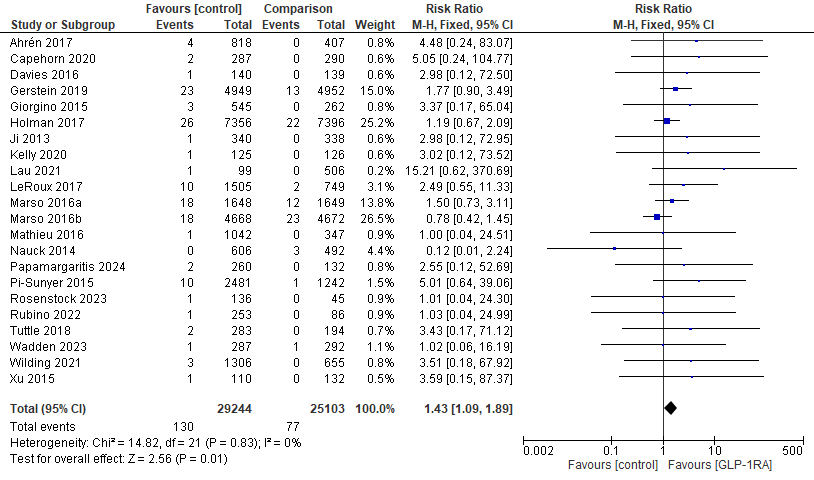
**

**Figure S6. Sensitivity Analysis Minimum 24-Weeks of GLP-1 RA versus control for comparison of pancreatitis incidence**

**
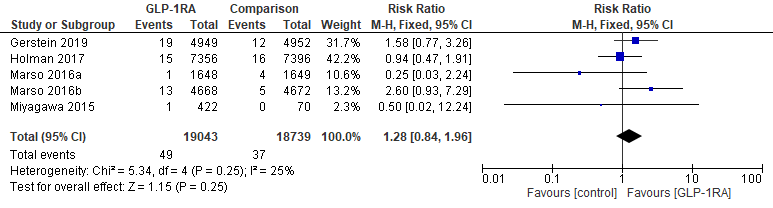
**

**Figure S7. Sensitivity Analysis Minimum 24-Weeks of GLP-1 RA versus control for comparison of pancreatic cancer incidence**
