# Supplementary material for: Evaluating the Rates of Pancreatitis and Pancreatic Cancer Among GLP‐1 Receptor Agonists: A Systematic Review and Meta‐Analysis of Randomised Controlled Trials
Source: Endocrinol Diabetes Metab. 2025 Sep 23;8(5):e70113. doi: 10.1002/edm2.70113 (PMC12457091; doi:10.1002/edm2.70113)
Supplement: Supplementary file 2 — Table S1: Cochrane Risk of Bias. Table S2: Summary of Cochrane Risk of Bias. [file EDM2-8-e70113-s002.docx]

| **Author,**  **Publication Year** | **Sequence Generation** | **Allocation concealment** | **Blinding of Partipants and personnel for All outcomes** | **Blinding of outcome assessors for All outcomes** | **Incomplete outcome data for All outcomes** | **Selective outcome reporting** | **Other sources of bias** |
| --- | --- | --- | --- | --- | --- | --- | --- |
| **Ahmann 2015** | **?** | **-** | **-** | **?** | **-** | **-** | **-** |
| **Ahren 2016** | **?** | **?** | **?** | **?** | **-** | **-** | **-** |
| **Ahrén 2017** | **-** | **-** | **-** | **-** | **-** | **-** | **-** |
| **Araki 2015** | **-** | **?** | **+** | **+** | **-** | **-** | **-** |
| **Armstrong 2016** | **-** | **-** | **-** | **-** | **-** | **-** | **-** |
| **Azar 2016** | **-** | **+** | **+** | **+** | **-** | **-** | **+** |
| **Bailey 2016** | **-** | **?** | **?** | **?** | **-** | **-** | **-** |
| **Buse 2009** | **+** | **+** | **+** | **+** | **-** | **?** | **+** |
| **Capehorn 2020** | **-** | **?** | **+** | **+** | **-** | **-** | **-** |
| **Chen 2024** | **-** | **-** | **-** | **-** | **-** | **-** | **+** |
| **Davies 2015** | **-** | **-** | **?** | **?** | **-** | **-** | **+** |
| **Davies 2016** | **-** | **?** | **-** | **?** | **-** | **-** | **-** |
| **Drucker 2008** | **?** | **?** | **+** | **+** | **-** | **-** | **-** |
| **Dungan 2014** | **-** | **+** | **+** | **+** | **?** | **-** | **+** |
| **Dungan 2016** | **?** | **?** | **?** | **?** | **-** | **-** | **-** |
| **Gerstein 2019** | **-** | **-** | **-** | **-** | **-** | **-** | **-** |
| **Giorgino 2015** | **-** | **?** | **+** | **+** | **-** | **-** | **-** |
| **Holman 2017** | **-** | **-** | **-** | **-** | **-** | **-** | **+** |
| **Inagaki 2016** | **?** | **?** | **?** | **+** | **+** | **-** | **+** |
| **Ji 2013** | **-** | **?** | **+** | **+** | **-** | **-** | **-** |
| **Kelly 2020** | **-** | **-** | **-** | **?** | **-** | **-** | **-** |
| **Klausen 2023** | **-** | **-** | **-** | **-** | **+** | **-** | **-** |
| **Klein 2014** | **?** | **?** | **-** | **-** | **-** | **-** | **-** |
| **Kuchay 2020** | **-** | **-** | **+** | **+** | **-** | **-** | **-** |
| **Lau 2021** | **-** | **-** | **?** | **?** | **-** | **-** | **+** |
| **LeRoux 2017** | **-** | **-** | **-** | **-** | **-** | **-** | **+** |
| **Marso 2016** | **-** | **-** | **-** | **-** | **-** | **-** | **-** |
| **Marso 2016** | **-** | **-** | **?** | **+** | **-** | **-** | **-** |
| **Mathieu 2016** | **-** | **?** | **?** | **?** | **-** | **-** | **+** |
| **Meier 2015** | **-** | **+** | **+** | **+** | **-** | **-** | **+** |
| **Miras 2019** | **-** | **-** | **-** | **-** | **-** | **-** | **-** |
| **Miyagawa 2015** | **-** | **+** | **+** | **+** | **-** | **-** | **-** |
| **Myat 2021** | **-** | **-** | **-** | **?** | **-** | **-** | **+** |
| **Nauck 2014** | **-** | **?** | **-** | **-** | **+** | **-** | **-** |
| **Nauck 2012** | **-** | **?** | **+** | **+** | **-** | **-** | **+** |
| **Papamargaritis 2024** | **-** | **-** | **+** | **+** | **+** | **-** | **+** |
| **Pi-Sunyer 2015** | **-** | **?** | **-** | **-** | **-** | **-** | **-** |
| **Pozzilli 2017** | **-** | **?** | **?** | **?** | **-** | **-** | **-** |
| **Pratley 2012** | **?** | **?** | **+** | **+** | **-** | **-** | **+** |
| **Pratley 2010** | **-** | **-** | **+** | **-** | **-** | **-** | **+** |
| **Retnakaran 2014** | **-** | **-** | **-** | **-** | **-** | **-** | **-** |
| **Rosenstock 2023** | **-** | **-** | **-** | **-** | **-** | **-** | **-** |
| **Rosenstock 2017** | **-** | **-** | **-** | **-** | **-** | **-** | **-** |
| **Rosenstock 2009** | **?** | **?** | **-** | **-** | **-** | **-** | **-** |
| **Rubino 2022** | **-** | **+** | **+** | **+** | **+** | **-** | **+** |
| **Santilli 2017** | **-** | **?** | **?** | **?** | **-** | **-** | **+** |
| **Seino 2010** | **?** | **?** | **?** | **?** | **-** | **-** | **-** |
| **Siskind 2018** | **-** | **-** | **+** | **+** | **-** | **-** | **+** |
| **Smits 2017** | **-** | **?** | **-** | **-** | **-** | **-** | **+** |
| **Sorli 2017** | **-** | **-** | **-** | **-** | **+** | **-** | **+** |
| **Tanaka 2015** | **?** | **?** | **+** | **+** | **-** | **-** | **+** |
| **Terauchi 2014** | **?** | **?** | **?** | **?** | **-** | **-** | **+** |
| **Tuttle 2018** | **-** | **+** | **+** | **+** | **-** | **-** | **-** |
| **Umpierrez 2011** | **-** | **?** | **?** | **?** | **-** | **-** | **-** |
| **vanRaalte 2016** | **-** | **?** | **?** | **?** | **-** | **-** | **+** |
| **Wadden 2013** | **-** | **-** | **-** | **-** | **-** | **-** | **-** |
| **Wadden 2023** | **-** | **?** | **-** | **-** | **-** | **-** | **?** |
| **Wägner 2019** | **-** | **?** | **-** | **-** | **-** | **-** | **+** |
| **Wilding 2021** | **-** | **?** | **-** | **-** | **-** | **-** | **+** |
| **Xu 2015** | **-** | **-** | **+** | **+** | **-** | **-** | **-** |
| **Zinman 2009** | **-** | **?** | **?** | **?** | **-** | **-** | **+** |
| **Zinman 2007** | **-** | **-** | **?** | **?** | **+** | **-** | **+** |

**Table S1: Cochrane Risk of Bias**

| **Author,**  **Publication Year** | **Sequence Generation** | **Allocation concealment** | **Blinding of Participants and personnel for All outcomes** | **Blinding of outcome assessors for All outcomes** | **Incomplete outcome data for All outcomes** | **Selective outcome reporting** | **Other sources of bias** |
| --- | --- | --- | --- | --- | --- | --- | --- |
| **Low Risk** | **52** | **28** | **27** | **25** | **58** | **65** | **36** |
| **Unclear** | **13** | **31** | **16** | **18** | **1** | **1** | **1** |
| **High Risk** | **1** | **7** | **23** | **23** | **7** | **0** | **29** |

**Table S2: Summary of Cochrane Risk of Bias**
